# SARS-CoV-2—Morphology, Transmission and Diagnosis during Pandemic, Review with Element of Meta-Analysis

**DOI:** 10.3390/jcm10091962

**Published:** 2021-05-03

**Authors:** Katarzyna Grudlewska-Buda, Natalia Wiktorczyk-Kapischke, Ewa Wałecka-Zacharska, Joanna Kwiecińska-Piróg, Katarzyna Buszko, Kamil Leis, Klaudia Juszczuk, Eugenia Gospodarek-Komkowska, Krzysztof Skowron

**Affiliations:** 1 Department of Microbiology, Ludwik Rydygier Collegium Medicum, Nicolaus Copernicus University in Toruń, 87-094 Bydgoszcz, Poland; katinkag@gazeta.pl (K.G.-B.); natalia12127@gmail.com (N.W.-K.); j.kwiecinska-pirog@cm.umk.pl (J.K.-P.); gospodareke@cm.um.pl (E.G.-K.); 2 Department of Food Hygiene and Consumer Health, Wrocław University of Environmental and Life Sciences, 50-375 Wrocław, Poland; ewa.walecka@upwr.edu.pl; 3 Department of Theoretical Foundations of Biomedical Science and Medical Informatics, Ludwik Rydygier Collegium Medicum, Nicolaus Copernicus University in Toruń, 87-067 Bydgoszcz, Poland; buszko@cm.umk.pl; 4 Faculty of Medicile, Ludwik Rydygier Collegium Medicum, Nicolaus Copernicus University in Toruń, 87-067 Bydgoszcz, Poland; kamil.leis13@gmail.com; 5 Clinic of General, Colorectal and Oncological Surgery, Dr. Jana Biziel University Hospital, No. 2 in Bydgoszcz, 75 Ujejskiego St., 85-168 Bydgoszcz, Poland; ontiunia@gmail.com

**Keywords:** SARS-CoV-2, COVID-19, pandemic, transmission route, laboratory diagnosis, childbirth

## Abstract

The outbreak of Coronavirus disease 2019 (COVID-19) is caused by severe acute respiratory syndrome (SARS) coronavirus 2 (SARS-CoV-2). Thus far, the virus has killed over 2,782,112 people and infected over 126,842,694 in the world (state 27 March 2021), resulting in a pandemic for humans. Based on the present data, SARS-CoV-2 transmission from animals to humans cannot be excluded. If mutations allowing breaking of the species barrier and enhancing transmissibility occurred, next changes in the SARS-CoV-2 genome, leading to easier spreading and greater pathogenicity, could happen. The environment and saliva might play an important role in virus transmission. Therefore, there is a need for strict regimes in terms of personal hygiene, including hand washing and surface disinfection. The presence of viral RNA is not an equivalent of active viral infection. The positive result of the RT-PCR method may represent either viral residues or infectious virus particles. RNA-based tests should not be used in patients after the decline of disease symptoms to confirm convalescence. It has been proposed to use the test based on viral, sub-genomic mRNA, or serological methods to find the immune response to infection. Vertical transmission of SARS-CoV-2 is still a little-known issue. In our review, we have prepared a meta-analysis of the transmission of SARS-CoV-2 from mother to child depending on the type of delivery. Our study indicated that the transmission of the virus from mother to child is rare, and the infection rate is not higher in the case of natural childbirth, breastfeeding, or contact with the mother. We hope that this review and meta-analysis will help to systemize knowledge about SARS-CoV-2 with an emphasis on diagnostic implications and transmission routes, in particular, mother-to-child transmission.

## 1. Introduction

SARS-CoV-2 (severe acute respiratory syndrome coronavirus 2) is a beta-coronavirus responsible for the COVID-19 disease [1]. It is the seventh coronavirus causing infection in humans, and the third, after SARS-CoV-1 and MERS, that triggered the epidemic and have pandemic potential [2,3,4,5,6]. The infection spreads mainly through the droplet route and can be asymptomatic, mild or acute, with fever, cough and running nose, and respiratory illness, which in some cases have a fatal outcome [7,8,9]. It is estimated that mortality affects 3–9% of all cases [1,10]

The first appearance of the pathogen was recorded in 2019 in Wuhan, China, most likely as a result of an adaptation of the virus transmitted from bats to humans. The official date is 17 November 2019. Over time, it spread to all continents, resulting in WHO (World Health Organization) announcing the pandemic state on 11 March 2020 [1,11,12]. An important factor in tracking the spread of coronavirus is the effective reproduction number of an infectious disease (R). Since many factors influence the value of the R factor, specialists interpret it with caution. Data, such as the number of deaths, hospitalized people, or positive tests for the virus, allow estimation of the virus spread. One of the mathematical models was developed by Arroyo et al. [13]. Provided that the percentage of detected cases is approximately constant, the model shows reliable estimates, even if not all COVID-19 cases are detected.

This review aimed to collect and systematize current data, hypotheses, and information related to the morphology, transmission, and diagnosis of SARS-CoV-2 based on the publications available in scientific databases. The second goal of the present review was meta-analysis to assess the risk of transmission SARS-CoV-2 from mother to child, depending on the type of delivery (vaginal birth vs. cesarean).

## 2. Taxonomy

According to the findings of the International Committee on Taxonomy of Viruses (ICTV), SARS-CoV-2 belongs to the *Sarbecovirus* subgenus, the B-type line of beta-coronavirus, the subfamily *Orthocoronavirinae*, the family *Coronaviridae* (which also includes the subfamily *Letovirinae*), and the order *Nidovirales* [5,14,15,16]. The *Coronaviridae* family is divided into 4 types: alpha-coronaviruses, beta-coronaviruses, gamma-coronaviruses, and delta-coronaviruses. Other representatives of the beta-coronavirus genus besides SARS-CoV-2 include SARS-CoV-1, MERS, HKU-1, HKU-4, HKU-5, HKU-9, OC43, BetaCov-1, and MurineCov. Together with six other viruses: MERS, SARS-CoV-1, 229E, HKU-1, NL63, and OC43, SARS-CoV-2 belongs to the group of coronaviruses (CoVs) pathogenic to humans (of which two belong to alpha-coronavirus and five to beta-coronavirus) [14,17,18,19].

## 3. Structure and Variants of SARS-CoV-2

Structurally, the viral units of SARS-CoV-2 are most reminiscent of RaTG13-coronavirus. Moreover, a high degree of protein E (100%), M (98.2%), N (96.7%), and S (90.4%) homology with CoVs isolated from Malayan pangolins (*Manis javanica*) was found. A comparison of the genomes suggests recombination between pangolin-CoV-like viruses with the bat-CoV-RaTG13-like virus [20]. SARS-CoV-2 probably acquired some mutations that allowed it to infect humans [21]. CoVs, like other RNA viruses, are highly variable. Such genetic variability can affect their pathogenicity, adaptation to new hosts, and cell or tissue tropism, leading to the emergence of infectious diseases with a previously unknown clinical course. The variability of CoVs results from the chemical structure of the genome (extremely labile RNA) and the characteristics of RNA-dependent RNA polymerase (which lacks repair mechanisms). During replication of CoVs, one mutation per every 10,000 nucleotides occurs [22]. It has been found that the SARS-CoV-1 genome is copied from several templates, which decreases the mutation rate [23]. We can suppose that the same mechanism is possible for SARS-CoV-2. Zhang et al. [24] identified in SARS-CoV-2 isolates several genomic regions of increased genetic variation. The similarity of the amino acid sequence within the spike glycoprotein is about 96–97%, while the identity with SARS-CoV-1 is about 80% [6,25]. The homology between SARS-CoV-2 and the Middle East respiratory syndrome coronavirus (MERS-CoV) is 50% [12]. The latest variants of SARS-CoV-2 are worrying as they have greater replication efficiency and transmission capacity and may be more lethal. The most famous variants of the coronavirus are British, Brazilian, and South African. The first significant change in the properties of SARS-CoV-2, as a result of a mutation located in the carboxy(C)-terminal region of the S1 domain, took place at the beginning of the pandemic in March/April 2020 (variant D614G) [26,27]. The changes in the amino acids of the spike protein increased the replication efficiency and transmission capacity of this virus. Due to these characteristics, the D614G variant has become dominant around the world. However, it did not lead to such severe consequences as those variants that have emerged recently [28]. British variant (VOC 202012/01 or B.1.1.7) was first identified in September 2020 in the south of the United Kingdom. This variant contains nine spike protein mutations (three deletions: 69H–70V, 145V; and six substitutions: N501Y, A570D, D614G, P681H, T716I, S982A, D1118H) [29]. Its spreading capacity is significantly higher. This variant is 25 to 40 percent more infectious than other forms of the SARS-CoV-2, which has contributed to increased incidence, hospitalization, and pressure on the healthcare system. The VOC 202012/0 variant is not only much more contagious, but also possibly more lethal, although these reports still require in-depth analysis [30]. South African variant (501Y.V2) was first identified in December 2020 in South Africa, where it is currently the most widespread variant. This mutation shares some similarities with the UK variant and is similarly more transferable. Thus far, there is no evidence that it causes more severe COVID-19 or is more fatal. Thus far, 31 countries, including Canada, Australia, Israel, and 10 European Union countries, have reported on 501Y.V2. The Brazilian variant (P.1 variant) has been already identified in Manaus, Amazonas state, north Brazil, and among travelers from several countries who have recently visited the area. Sequencing studies showed that the variant appeared in July 2020. The P.1 variant contains 17 mutations, including three in the spike protein (K417T, E484K, and N501Y) associated with increased binding to the human ACE2 receptor [31].

### 3.1. Morphology

CoVs are RNA viruses and have the largest genomes (26–32 kb) among all RNA virus families. Each viral transcript has a 5′-cap structure and a 3′ poly (A) tail [32]. The diameter of a single virion is 50–200 nm [14]. Most often, the virion of SARS-CoV-2 has a spherical form, but also pleomorphic and oval shapes occur. The viral envelope is built of three proteins: the S-spike protein that forms the peplomers and gives the virus a characteristic crown shape, M-membrane protein, and E-envelope protein, which provide the ring structure [2,16]. There is also a fourth protein, N-nucleocapsid protein, a phosphoprotein, which is a structural component of the nucleocapsid [4,6,14,16,33].

The S protein belongs to the group I fusion glycoproteins. It is characterized by a homotrimeric structure with a single upper and two lower conformations [25,34,35,36]. The identity of the amino acid sequence of the S protein between SARS-CoV-1 and SARS-CoV-2 is about 75.5% [37]. The spike is composed of two subunits: the N′-terminal S1 and the C′-terminal S2, responsible for the association with the host cell and virion endocytosis, respectively. Between them is a 4-amino-acid region, involved in the cleavage of furin protein during biosynthesis, which distinguishes SARS-CoV-2 from SARS-CoV-1 [18,25,38,39,40]. The S1 subunit contains a 200-amino-acid domain binding receptor (RBD) known as the CTD-C-terminal domain [3]. The S1 and S2 SARS-CoV-2 subunits have approximately 64% and 90% similarity to the analogous subunits in SARS-CoV-1, respectively [25,35,41].

A structural E protein participates in the formation of new virions that are mostly built from the host cell material. It is characterized by a large amount of valine and has a lower guanine-cytosine percentage than the analogous structural protein SARS-CoV-1 [14]. It probably still has a more similar structure to the analogous SARS-CoV-1 than other proteins, in particular the M protein [42].

### 3.2. Genome

The virus has one uniform strand of positive-sense RNA with a linear shape and a length of 29,903 bp. [2,5,6,14,16]. Sequence analysis of SARS-CoV-2 isolates revealed 14 open-reading frames encoding 29 proteins [43]. The 5′ terminus of the genome contains ORF1ab and ORF1a genes. ORF1ab is the largest gene and encodes the pp1ab protein that contains 15 non-structural proteins (nsp1-nsp10 and nsp12–nsp16). In CoVs, nsp16, in conjunction with nsp10, methylates the 5′-end of virally encoded mRNAs to mimic cellular mRNAs, thereby protecting the virus from host innate immune restriction [14].

Within the SARS-CoV-2 genome, one deletion at the 3′-end region (10 nt) and 2 deletions in the 1ab polyprotein (3 and 24 nt) were identified. Based on the previous analyses, 93 mutations including 29 missenses in 1ab polyprotein, 8 within the structural S protein, 4 within the structural N protein, and 1 in the structural M protein, were also found. Researchers point out the role of mutations in the spike protein, as it is a key element of the initial infection phase, and changes in an amino acid sequence result in a new glycoprotein conformation and newly acquired antigenic traits [44,45]. To date, research on the localization of amino acids involved in structural changes of the SARS-CoV-2 spike surface glycoprotein structure is not published. The identification of these amino acids is of significance and should be investigated by further studies.

In the SARS-CoV-2 genome, also polymorphisms of the amino acid sequence (positions 203 and 204) have been reported. The findings suggest homologous recombination as a mechanism for increased viral fitness and adaptation of SARS-CoV-2 to the human population [45].

## 4. Receptors for SARS-CoV-2 Entry and Replication Cycle

**Figure 1:**
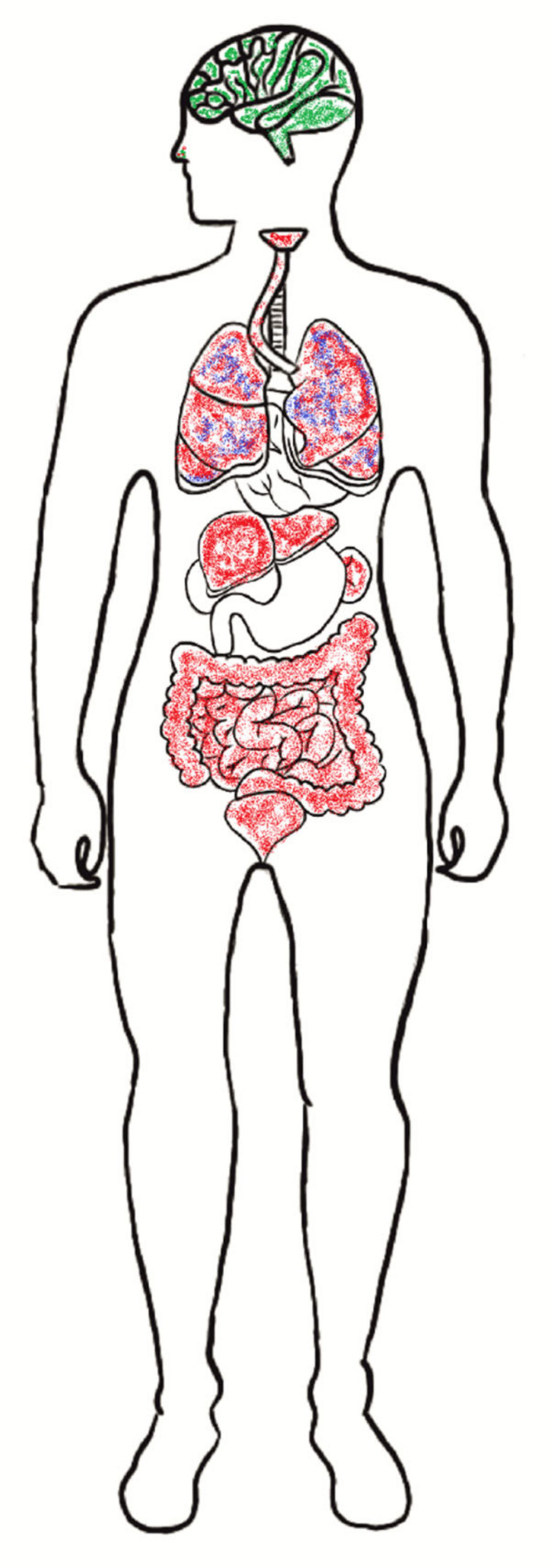
Expression of known and possible receptors for SARS-CoV-2; red color—expression of ACE-2, green color—expression of DPP4, blue color—expression of NRP1.

SARS-CoV-2 is an intracellular microorganism that replicates and spreads through the machinery of the host cell. CoV entry into host cells is mediated by the transmembrane S glycoprotein [46,47]. Receptors and proteases on the surface of the host cells play an important role in the pathogenesis of SARS-CoV-2. The best-known receptor for SARS-CoV-2 is angiotensin-converting enzyme 2 (ACE2) [48]. Generally, ACE2 is a zinc metalloprotease (carboxypeptidase). It is a homolog of dipeptidase angiotensin-converting enzyme (ACE), but with different substrate specificity. The binding affinities reported by different groups were varied [18,25]. Wrapp et al. [49] showed that ACE2 bound to the SARS-CoV-2 virus S protein ~10- to 20-fold stronger than to SARS-CoV-1. However, Walls et al. [25] and Nguyen et al. [50] showed that SARS-CoV-2 has a higher binding affinity, but the difference between ACE and ACE2 is relatively small. It can be hypothesized that ACE2 gene polymorphism and its expression influence the SARS-CoV-2 susceptibility and the COVID-19 disease outcome. The presence of variants: E23K, H378R, I21V, K26R, N64K, S19P, T27A, T92I, and Q102P may result in easy infection of cells. In turn, variants: D38V, D355N, D509Y, E35K, E37K, F72V, G326E, G352V, H34R, K31R, K68E, M62V, N33I, N51S, Q388L, Y50F, and Y83H hinder virions’ penetration [51]. Li et al. [52] showed that ACE2 is expressed in various human tissues in addition to the lungs, indicating that SARS-CoV-2 may also infect other tissues (Figure 1). An alternate entry receptor for the virus is a transmembrane glycoprotein CD147, commonly known as basic immunoglobulin (Basigin) or extracellular matrix metalloproteinase inducer (EMMPRIN) [53,54]. Qiao et al. [55] detected the expressions of CD147 in human and mouse brain cell lines. Wang et al. [54] performed surface plasmon resonance (SPR) and enzyme-linked immunosorbent assay (ELISA) and showed an interaction between CD147 and spike(RBD). Moreover, an electron microscope demonstrated virions in Vero E6 cells, lung, and kidney tissues. In contrast, Shilts et al. [56] indicated that the recombinant forms of the SARS-CoV-2 spike do not interact with CD147 expressed on the surface of human cells. These reports question the role of this receptor in the pathogenesis of SARS-CoV-2. Due to conflicting reports, further studies are needed to confirm or exclude CD147 as the entry receptor during SARS-CoV-2 infection. Another receptor to facilitate SARS-CoV-2 entry is neuropilin (NRP). Neuropilin is a transmembrane glycoprotein active in neurons. There are two forms of neuropilins, NRP-1 and NRP-2, with 44% similarity at the amino acid level [57]. Cantuti-Castelvetri et al. [58] demonstrated NRP-1 expression in almost all pulmonary and olfactory cells, with the highest expression in endothelial cells. This cell surface receptor has a crucial role in angiogenesis, tumor progression, viral entry, axonal guidance, and immune function. Daily et al. [59] indicated that the CendR (C-end rule) motif in SARS-CoV-2 S1 protein bound to NRP-1 in cell culture study. However, no impact was found on cell surface attachment, this interaction promotes entry and infection by SARS-CoV-2. The alternative receptor for SARS-CoV-2 is dipeptidyl peptidase 4 (DPP4), also known as CD26. This is an ectopeptidase expressed on different types of cells, including the immune system, kidneys, lungs, smooth muscle, liver, and capillaries. Vankadari et al. [60] and Qi et al. [61] revealed an interaction between DPP4 and spike glycoprotein of SARS-CoV-2. However, these studies were only predictive-based preliminary research, and direct involvement of DPP4 in SARS-CoV-2 infection merits further investigation explanation. Based on studies on other CoVs (HCoV-229E, SARS-CoV) it can be assumed that SARS-CoV-2 uses other receptors, i.e., ANPEP (alanyl aminopeptidase), ENPEP (glutamyl aminopeptidase), and AGTR2 (angiotensin II receptor type 2) [61,62]. Nonetheless, there is strong evidence only for ACE-2 as an essential functional receptor protein [35].

Structural modeling suggests that the ACE2-B0AT1 complex can bind two S proteins simultaneously, providing important clues to the molecular basis for coronavirus recognition and infection [36]. Its higher expression was found in men than in women, which possibly explains the higher sensitivity of men to SARS-CoV-2 [16,36,40,49,51,63,64]. B0AT1 has also been shown to interact with another coronavirus receptor, aminopeptidase N (APN or CD13) [36]. These findings suggest that B0AT1 may play a regulatory role in the enteric infections of some CoVs.

**Figure 2:**
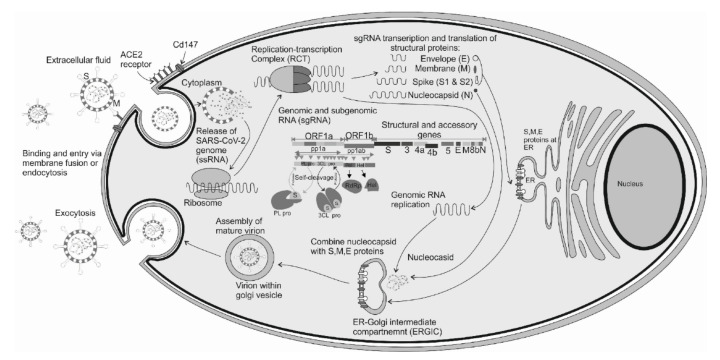
Mechanism of SARS-CoV-2 infection of human cells via the interaction of spike glycoprotein, the ACE2 receptor protein, and the CD147 receptor. Genomic structure and proteins encoded by SARS-CoV-2.

Upon cell entry, the ectodomain of SARS-CoV-2 RBD, located at the S1 subunit of the S protein forms a complex with a glycolysated region (sites 5 and 7) of the hACE2 extracellular peptide domain (Figure 2).

SARS-CoV can use the endosomal cysteine proteases cathepsin B and L (CatB/L) and the cellular serine protease TMPRSS2 for S protein priming, and inhibition of both proteases is required for a robust blockade of viral entry [65,66]. Ou et al. [35] demonstrated in the cell-line study that SARS-CoV-2 enters mainly through endocytosis, and cathepsin L is critical for the entry [28]. Hoffman et al. [67] found that multibasic S1/S2 site in the spike protein of SARS-CoV-2 is indispensable for efficient proteolytic cleavage of the spike protein in cells with expression of TMPRSS2. The fragmented spike protein is responsible for inhibiting the immune response [12,66,67,68]. The M protein also participates in the attachment and entry into the host cell [42].

After viral structures enter the cell by endocytosis, replication occurs. Due to intensified recombination events, this process distinguishes SARS-CoV-2 from other RNA viruses [14,18,35]. In the acidic environment, the further division of the S protein by L cathepsin occurs. As a consequence, the genetic material of SARS-CoV-2 is released to the cytoplasm [68]. The next step is a reverse transcription (Figure 2). Transcription processes occur with the participation of the vesicular replication–transcription complex. A negatively charged strand of RNA is formed, which is a matrix for mRNA production involving the host ribosome [7]. Transcription ends between ORFs in so-called regulatory sequences.

Next, ORF1a and ORF1ab are translated producing pp1a and pp1ab polyproteins (from about 2/3 of the genetic material of the virus) (Figure 2) [7,68,69,70]. Produced pp1a and pp1ab polypeptides then undergo modifications by the main protease, papain-like proteases (PL1pro, PL2pro), and 3CLpro proteases [7,71]. PLpro is an essential enzyme required for processing viral polyproteins to generate a functional replicase complex and enable viral spread. The sequence identity between SARS-CoV-1 and SARS-CoV-2 PLpro is approximately 83%. Nevertheless, the SARS-CoV-2 PLpro exhibits different host substrate preferences [72]. In particular, SCoV2-PLpro preferentially cleaves the ubiquitin-like protein ISG15, whereas SCoV-PLpro predominantly targets ubiquitin chains. These results highlight a dual therapeutic strategy in which targeting of SCoV2-PLpro can suppress SARS-CoV-2 infection and promote anti-viral immunity [73]. As a consequence, all nsp proteins are formed. In the case of structural proteins, translation is mediated by the endoplasmic reticulum, and the matrix is a single guide RNA (sgRNA) [7,68,74].

Once all viral components are produced and combined with the participation of the M protein, an endoplasmic reticulum-Golgi intermediate compartment—ERGIC is formed. After assembly, enveloped virions and exocytosis occur [7,68]. The presence of the pathogen stimulates the immune response manifested by an increased concentration of interferon and inflammatory cytokines [75,76].

Based on the analysis of urine and feces samples, it is believed that replication can occur, in addition to the respiratory tract, in the lumen of the gastrointestinal tract and the urinary tract [77,78]. This possibility is confirmed by the presence of hACE receptors that are the target of viral infection in the renal tissue and enterocytes (progenitor or Apo A1 +). In the case of renal parenchyma, the virus multiplies in organoids (e.g., tubular cells) and enterocytes in the apical or basolateral part of the organoids. Butowt et al. [79] indicated that the probable site of enhanced SARS-CoV-2 binding is the olfactory epithelium of the nasal cavity. Many types of non-neuronal cells present in the olfactory epithelium have two host receptors, the ACE2 and TMPRSS2 proteases, which facilitate SARS-CoV-2 binding, replication, and accumulation.

An important step in understanding and further researching the virus was in vitro culturing. Harcourt et al. [38] examined the capacity of SARS-CoV-2 to infect and replicate in several common primate and human cell lines, including human adenocarcinoma cells (A549), human liver cells (HUH7.0), and human embryonic kidney cells (HEK-293T). In addition, Vero E6 and Vero CCL81 cells and the big brown bat kidney cell line (EFK3B) were tested. Harcourt et al. [38] passaged the virus in Vero CCL-81 cells and titrated by determining the 50% tissue culture infectious dose (TCID_50_). Titers were 8.65 × 10^6^ TCID_50_/mL for the third passage and 7.65 × 10^6^ TCID_50_/mL for the fourth passage. These results are consistent with previous reports for SARS-CoV and MERS-CoV, which suggested similar replication dynamics between other coronavirus strains. It is believed that other cell lines and systems used in in vitro virus propagation, including MDCK, HeLa, HEP-2, MRC-5 cells, and embryonated eggs, are unlikely to support SARS-CoV-2 replication. Moreover, SARS-CoV-2 did not replicate in bat EFK3B cells, which are susceptible to MERS-CoV. Based on the research conducted so far, it can be assumed that cell lines display similar susceptibility to SARS-CoV-2 and SARS-CoV-1 [80].

## 5. Physical and Chemical Properties

SARS-CoV-2 is characterized by relatively low resistance to high temperatures. It survives at 56 °C only for 30 min. It is also sensitive to UV radiation, ethanol, ether (both in 75% concentration), and preparations containing such compounds as peracetic acid, chloroform, and chlorine [7,16]. Additionally, it shows resistance to chlorhexidine. Due to the high identity with SARS-CoV-1, SARS-CoV-2 may show similar chemical and physical properties. SARS-CoV-1 remains alive for about 5 days at 50% humidity and a temperature range of 22–25 °C. Moriyama et al. [81] showed that low humidity and temperature increase the lifespan of the virus in the aerosol. Chin et al. [82] reported that SARS-CoV-2 was highly stable at 4 °C but sensitive to heat. At 4 °C, there was only around a 0.7 log-unit reduction of infectious titer on the 14th day. The virus survival time was shortened to 5 min as the incubation temperature increased to 70 °C. This virus shows stability over a wide pH range [3,4,5,6,7,8,9,10] at room temperature [82].

### 5.1. SARS-CoV-2—Stability in Air and on Surface

The main transmission route of SARS-CoV-2 is droplet [83]. Air can be an important route of SARS-CoV-2 virus transmission, especially in hospitals, shops, schools, and public transport [84]. Particular attention should be paid to closed rooms where the density of people is high and proper ventilation is limited [85,86]. According to WHO [86], transmission by droplets may occur in the case of close contact with an infected person, i.e., 1 m. It should be noted that infection occurs when aerosols contain an infectious dose of the virus (which currently is unknown) [86]. van Doremalen et al. [87] showed that the virus remained stable up to 3 h in a closed rotating drum (infectious titer—10^3.5^ TCID_50_/L, reduction to 10^2.7^ TCID50/L; 21–23 °C, 65% relative humidity). In turn, Fears et al. [88] found the viable SARS-CoV-2 virus in 16-L chambers after 16 h (a small but constant fraction of SARS-CoV-2). Several studies have been conducted to assess the presence of SARS-CoV-2 in the air [89,90]. The transmission of SARS-CoV-2 in buses via aerosol has been noted [89]. In turn, Jiang et al. [90] tested air samples collected at the hospital in zones designated as high-risk area, middle-risk area, and low-risk area. Jiang et al. [90] assessed the air by natural sedimentation and microbial air sampler (MAS-100 ECO, 100 L/minute). Researchers showed that one air sample (1/28; 3.57%) was positive for SARS-CoV-2 virus RNA (collected in high-risk areas (isolation ward with intensive care patients)) [90]. Guo et al. [91] revealed that 35% (14/40) and 12.5% (2/16) of samples from the intensive care unit and general COVID-19 ward, respectively, were positive for SARS-CoV-2 RNA. There have also been studies that showed no evidence of viral RNA in the air samples of hospital rooms [92,93,94]. Faridi et al. [93] tested the air in the rooms of patients with confirmed COVID-19 in the largest hospital in Iran. Ten air samples were collected using sterile standard midget impingers. All air samples (taken 2 to 5 m from the beds of patients with COVID-19) were negative [93]. It should be emphasized that the data mentioned above were based on the use of the RT-PCR technique, which does not prove the detection of viable viral particles.

Due to the stability of the virus on various surfaces, abiotic surfaces are also a source of nosocomial infections and can contribute to the spread of SARS-CoV-2 [90]. The stability of the virus directly affects the transmission, since its particles remain viable for a limited time after excretion by the host [95]. Van Doremalen et al. [87] demonstrated the SARS-CoV-2 stability up to 4 h on the surface of copper, 24 h on the surface of the carton, and up to 2–3 days on plastic and stainless steel. Jiang et al. [90] studying surface samples from various hospital wards, showed that only one surface sample from the nursing station (1/130, 0.77%) was positive for SARS-CoV-2. Ong et al. [92], in Singapore, took surface samples from COVID-19 patients (bedrooms before and after routine cleaning). Before routine cleaning, positive results were obtained for 87% (13/15) samples from the seats in the rooms (including exhaust fans) and 60% (3/5) of toilet samples (toilet bowl, sink, and door handle). After routine cleaning of the room, only one swab from the shoe surface was positive [92]. The risk of transfer from contaminated footwear is probably low, as evidenced by the negative results of tests carried out in the hallway and the corridor [92]. An important aspect is also the contamination of paper documents or paper banknotes with the SARS-CoV-2 virus. Chin et al. [82] showed that no infectious virus particles could be recovered from prints and tissue paper after 3 h, but the virus persisted for up to two days on the surface of wood and fabric. The virus was also detected on the banknote and glass (after 4 days), stainless steel, and plastic (after 7 days), as well as on the external surface of the surgical mask (after 7 days) [80]. Van Doremalen et al. [96] showed that despite comparable stability, the lifetime of SARS-CoV-2 was longer than SARS-CoV-1. SARS-CoV-2 can remain stable and infectious both in the aerosol and on the surface [96]. Viable virus in all surface and aerosol samples was quantified by end-point titration on Vero E6 cells as described previously [97].

Significant environmental pollution by COVID-19 patients’ respiratory droplets and fecal excretions [98,99] suggest that the environment is a potential means of virus transmission. Therefore, there is a need for strict regimes in terms of personal hygiene, including hand washing and environmental disinfection, as well as reducing the density of people in closed rooms and the use of protective masks.

### 5.2. Methods of Elimination

To minimize the risk of infection by contact with a contaminated surface, proper disinfection procedures should be implemented [100]. Information on the stability of the virus on various surfaces is crucial in the assessment of each disinfection process [101]. Alcohol-based disinfectants (ethanol, propan-2-ol, propan-1-ol) have been shown to significantly reduce the infectivity of enveloped viruses, such as SARS-CoV-2, at concentrations of 70–80% within one minute [100,101]. However, ethanol has not yet been approved by BPR (Biocidal Products Regulation), so its use is transitional [77]. Currently, biocidal products with virucidal activity are recognized as effective against SARS-CoV-2. This also applies to products used as hand and skin disinfectants that have limited virucidal activity or activity only against enveloped viruses [100]. The US EPA (the United States Environmental Protection Agency) has published an extensive list of chemicals effective against SARS-CoV-2, including quaternary ammonium compounds, peroxygen compounds, sodium hypochlorite, alcohols, and organic acids [102].

Disinfection and cleaning of frequently touched surfaces such as doors, toilets, desks, and sinks should be carried out using appropriate disinfectants. Disinfectants with the addition of 62–71% ethanol or 0.1% sodium hypochlorite have been shown to reduce surface contamination with the coronavirus within one minute of exposure [101]. Ozone water is widely used as a disinfectant for water and wastewater due to its bactericidal properties. However, EPA does not recommend the use of ozone water for surface disinfection and coronavirus contaminated water [102]. Darnell et al. [103] showed that CoVs are inactivated by exposure to ultraviolet C (UV-C) light within 15 min. The D_90_ value for various species of CoVs ranges from 7 to 241 J/m^2^, with an average value of 67 J/m^2^ showing the SARS-CoV-2 susceptibility to UV-C [104]. ASHRAE (American Society of Heating, Refrigerating and Air-Conditioning Engineers) recommends disinfection with ultraviolet radiation as one of the strategies for preventing SARS-CoV-2 transmission [105]. One method to prevent air movement in virus-infected sections (including hospital departments), and thereby the virus spread is a careful regulation and control of air-conditioning systems (under vacuum) [106]. Moreover, MRIGlobal conducted tests and evaluation of the room-wide air disinfection system using radiant catalytic ionization (ActivTek/RCI ActivePure). The study showed a significant reduction of the SARS-CoV-2 virus on the steel coupons in 3- and 6-h tests, with results of 93.27% and 97.95%, respectively.

## 6. Possibility of Transmission from Animals to Humans

CoVs can cause disease in domestic as well as wild animals, but in most cases, infections remain subclinical. The animal species that can be infected with CoVs include horses, camels, cattle, swine, dogs, cats, rodents, birds, ferrets, minks, bats, rabbits, snakes, and various other wild animals. Most investigations confirmed, like in the case of SARS-CoV-1 and MERS, the bat ware reservoir of SARS-CoV-2 [20,107].

At this point, the possibility of human transmission of the virus to other animals, especially pets, is in question. Such a possibility could play a significant role in the spread of the virus and constitute a potential route of human-to-human transmission if animals were found to be asymptomatic virus carriers. To date, there are few reports on the isolation of SARS-CoV-2 from animals, but the topic is being studied more.

**Figure 3:**
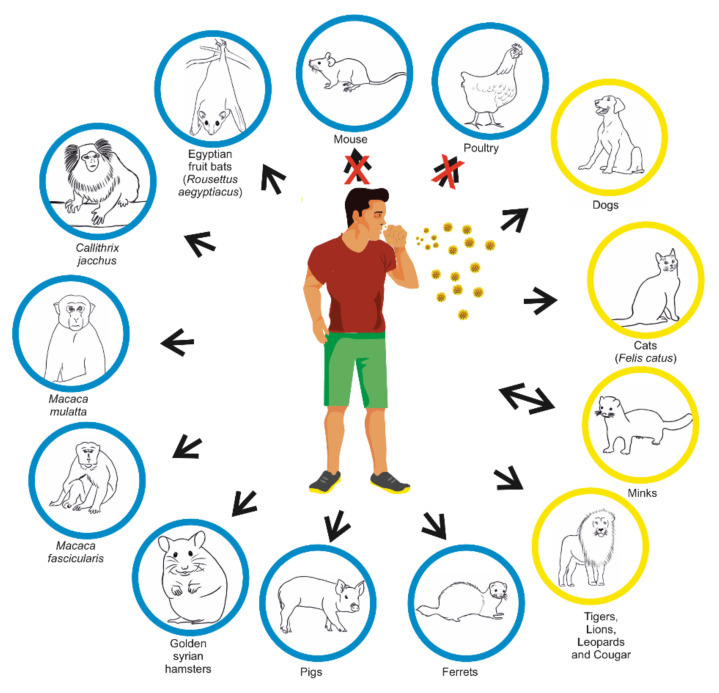
Possible transmission of SARS-CoV-2 from human to animals. Natural infection of animals—orange circle, experimental infection of animals—blue circle [116,117,118,119].

Natural infection with SARS-CoV-2 has been documented in several animals including pet cats and dogs, zoo felids, and mustelids (Figure 3) [108,109,110,111]. The United States Department of Agriculture (USDA) and the CDC reported the first confirmed cases of COVID-19 in two pet cats with mild respiratory illness in New York [8]. The cats had close contact with people confirmed or suspected to have COVID-19, suggesting human-to-cat spread. Cats have shown clinical signs of disease. Additionally, Zhang et al.’s [111] data confirmed cats’ infection with SARS-CoV-2. They described serum antibody dynamics in cats using indirect ELISA with the SARS-CoV-2 RBD protein. The cats showed general symptoms, then gastrointestinal and respiratory signs similar to those observed in humans, but no severe disease or death was observed [109,110]. Shi et al. [110] found that ferrets and cats are highly susceptible to SARS-CoV-2, while dogs are less sensitive. This data suggest that dogs can get infected, but might not spread the virus to other dogs as easily as cats and ferrets [110]. Schlottau et al. [20] observed that pigs and chickens could not be infected intranasally by SARS-CoV-2. The first farmed species infected with SARS-CoV-2 were minks. The first reports come from the Netherlands, Denmark, the US, and Spain [112,113]. Munnink et al. [112] used whole-genome sequencing showing that SARS-CoV-2 may spread from mink to human. SARS-CoV-2 infection in farmed mink was characterized by respiratory disease and increased mortality rate. Gortázar et al. [114] found SARS-CoV-2 RNA in swab samples from 6 of 71 ferrets in Ciudad Real province, central Spain, indicating a possible human–ferret transmission. The ferret is a suitable animal model for asymptomatic or mild SARS-CoV-2 infections in humans and other susceptible animals due to the humoral immune response reported in these animals [115]. However, further studies are needed to elucidate the SARS-CoV-2 impact on different animals [8].

## 7. Spreading of SARS-CoV-2 and Clinical Manifestation of COVID-19

Human atomization of viruses arises from the coughing or sneezing of an infected person, producing droplets (>5 μm) and aerosols (<5 μm) containing viruses. Virus transmission between people occurs via direct/indirect contact and air. Large droplets settle mainly in the air, contaminating people/objects while aerosols are effectively dispersed in the air. Inhaled airborne viruses accumulate directly in human airways [9,14,120,121]. Certain persons called “super spreaders” produce many more aerosol particles than others. The diameters of these particles are in the micron range. These particles are too small to settle because of gravity, but they are carried by air currents and dispersed by diffusion and air turbulence [122]. Guenther et al. [122] reported a transmission cluster in a German meat processing plant and provided data suggesting that environmental conditions promoted viral transmission from a single index case to more than 60% of co-workers within a distance of 8 m. This work indicates that a physical distance of 2 m does not suffice to prevent the transmission of SARS-CoV-2 [122]. Zhang et al. [121] indicated the possibility of both indoor and outdoor air transmission of the virus. Morawska and Cao [84] demonstrated that the SARS-CoV-2 virus, like the SARS-CoV-1 virus, can be transmitted by the air. Thus far, however, no case of SARS-CoV-2 infection by the air has been reported.

Infection can also occur through direct contact with an infected person (e.g., a handshake) and contact with objects contaminated by SARS-CoV-2 trapped in saliva or other discharges from the respiratory tract (some researchers, however, question this path of infection). Viral load in saliva ranges from 9.9 × 10^2^ to 1.2 × 10^8^ copies/mL and is the highest during the first week of symptom onset and then gradually declined. At initial screening, viral load and positivity rates were comparable to nasopharyngeal swabs. To et al. [123,124] indicated that salivary loads were five times higher than nasopharyngeal swabs. Until now, the infectious dose for COVID-19 is unknown, but most probably the value of 10^8^ RNA copies per 1 mL of saliva is enough to begin the infection. This transmission route seems to be more effective than in the case of MERS-CoV infection [125].

There is an increasing number of studies that identified a live virus in a stool sample from a patient [126]. Due to the low infectious dose of the SARS-CoV-1 virus [127], it is thought that the fecal–oral route may be one way of its transmission. This route seems to be important in household transmission. This increases the risk of COVID-19 spread, especially in areas with low hygiene. However, to determine if the virus is able to spread such a way, further research with a larger number of patients assessing the virus viability and fecal titers is needed.

There have also been reports on virus culturing from wastewater samples [128,129] suggesting the possibility of a water-borne transmission route. People who show no symptoms of COVID-19 can also be a source of infection, but there is a higher risk of transmission for a more susceptible person.

All three highly pathogenic CoVs can be transmitted from human to human. This route of transmission occurred in 57.6% of the total COVID-19 confirmed cases in Beijing, China [130,131]. Transmission between relatives occurred in only 13–21% of MERS-CoV cases and 22–39% of SARS-CoV-1 cases [132]. In our opinion, the predominance of household transmission for SARS-CoV-2 is probably related to the lower mortality rate for this virus in comparison to MERS-CoV. A much higher percentage of patients infected by MERS-CoV required intensive care unit administration (53–89 for MERS-CoV vs. 24 for SARS-CoV-2) [133].

All highly pathogenic human CoVs are responsible for infections, especially in older adults (>60 years old) with co-existing diseases. Of the 281 confirmed MERS-CoV infections, 160 (56.9%) involved comorbidities (diabetes mellitus, hypertension, ischemic heart disease, congestive heart failure, end-stage renal disease, and chronic kidney disease) [134,135]. Similar conclusions were obtained by Guan et al. [135] from an analysis of risk factors in 1590 patients with COVID-19 in China. The most common comorbidities in COVID-19 were: cardiovascular diseases, hypertension, and diabetes. A greater number of comorbidities was correlated with poorer clinical outcomes. The higher prevalent comorbidity was observed in patients with two or more co-existing diseases (1.61–4.17) than among patients with one comorbidity (1.16–2.77).

The probability of infection with SARS-CoV-2 applies to people of all ages. According to current studies, the majority of the COVID-19 patients were between 36 and 65 years old, indicating this group as the most susceptible to SARS-CoV-2 worldwide. The Chinese Center for Disease Control and Prevention [6] published a data report, which summarized the epidemiological characteristics of the COVID-19 outbreak in China. Out of the 72,314 cases investigated, 44,672 were diagnosed, and the distribution characteristics of the confirmed COVID-19 cases were as follows: 87% patients between 30–79 years old, 1% patients under 9 years old, 1% children between 10–19 years old, and 3% people of 80 years old or above. The overall fatality rate was 2.3%, while in severe cases the percentage reached up to 49%. The fatality rates varied for patients with underlying diseases—cardiovascular disease (10.5%), diabetes (5.6%), chronic respiratory diseases (6.3%), hypertension (6.0%), and cancer (5.6%) [6,43]. Nonetheless, severe symptoms are more frequent in the group of older people. This may be associated with the higher expression of the ACE-2 receptor induced by hypertension, which is also intensified by long-term pharmacotherapy with sartans [136]. Another concept is the occurrence of a phenomenon known as antibody-dependent enhancement, developed as a result of the contact with related pathogens in the past. Cancer patients, especially lung cancer, are also thought to be more susceptible to infection. Furthermore, other comorbidities, mainly diabetes, obesity, kidney, nervous, respiratory, and cardiovascular diseases are a predisposing factor. Infections in such patients are also more severe compared to immunocompetent individuals [137,138,139].

The incubation period for all highly pathogenic CoVs is similar and is estimated as 5 days for SARS-CoV-2 and SARS-CoV-1, and between 5 and 7 days for MERS-CoV [140]. The maximum incubation period of SARS-CoV-2 is 14 days, but most often symptoms appear within 3–7 days. The longest and the shortest documented interval between SARS-CoV-2 infection and clinical manifestation was 12.5 and 2 days, respectively.

The clinical spectrum of SARS-CoV-2 infection is broad, ranging from asymptomatic infection to flu-like illness or viral pneumonia. Fever is usually associated with severe dyspnea, respiratory distress, tachypnea (>30 breaths/min), and hypoxia (SpO2 < 90% on room air). The fever symptom must be interpreted carefully, as even in severe forms of the disease, it can be moderate or even absent. The diagnosis of Acute Respiratory Distress Syndrome (ARDS) requires clinical and ventilatory criteria. This syndrome is suggestive of a serious new-onset respiratory failure or for worsening of an already identified respiratory picture. Different forms of ARDS are distinguished based on the degree of hypoxia [141]. The clinical spectrum of COVID-19 is summarized in Table 1.

Furthermore, clinical observations indicate perfusion loss, caused by in situ thrombosis, as the primary mechanism of hypoxaemia in early COVID-19 respiratory failure. CT image is usually characterized by ground-glassing opacity and consolidation [143,144,145]. In addition to these symptoms, hemoptysis, moist rales, change in volume of voice tremor, and decrease in breathing noise may occur [43]. An indicator of the disease stage may be the level of interferon-alpha, interleukin-6, and chemokines: CCL5, CXCL8, and CXCL10 [70]. The factors predisposing the acute COVID-19 form development are the APOE E4 genotype, associated with Alzheimer’s disease or dementia, as well as the infection with the G614 strain, known as D614G (substitution of aspartate to glycine at position 614 in the amino acid sequence of the spike protein) [146,147,148,149,150,151]. In the case of pneumonia, alveoli are damaged with the possibility of hyaline deposits and infiltration of macrophages and mononuclear cells [77]. There may also be a so-called cytokine storm, with massive infiltration of interleukin 6 produced by leukocytes. In addition, interleukin 1-beta, interleukin 12, interleukin 18, interferon-alpha, interferon-gamma, TNF-alpha, and many chemokines are released. Such hyper-reactivity causes auto-aggression resulting in serious damage to the pulmonary parenchyma and other organs, and often the patient’s death [7,70].

In addition to respiratory system involvement, COVID-19 disease may contribute to intrarenal hemorrhage, acute heart damage, neuronal damage, lymph node necrosis, hepatomegaly, and spleen atrophy. There is also often a disturbed sense of taste and smell as a consequence of the olfactory epithelium invasion. In turn, digestive tract involvement contributes, among others, to diarrhea and vomiting. In their analyses, Xiao et al. [152] have observed intracellular staining of viral nucleocapsid protein in gastric, duodenal, and rectal epithelia. Although viral RNA was also detected in esophageal mucous tissue, the absence of viral nucleocapsid protein staining in the esophageal mucosa indicates low viral infection in the esophageal mucosa. Hence, it can be assumed that SARS-CoV-2 infects these gastrointestinal glandular epithelial cells [43,77,138,153,154,155,156]. In rare cases, SARS-CoV-2 viremia may occur, a condition in which virions are found in the blood of a sick person [76]. Franchini et al. [157] described the case of a man with the acquired type A haemophilia, re-developed as a result of virus infection. SARS-CoV-2 infection may also alter the mental status of geriatric patients (confusion, eating disorder, psychomotor agitation) without symptoms from other systems [158]. More SARS-CoV-2 may affect the central nervous system. Moriguchi et al. [159] have reported on encephalitis and meningitis in a 24-year-old man. In the beginning, the man had a headache and fever, then disturbed consciousness, neck stiffness, and epileptic seizures appeared. The test for SARS-CoV-2 from the respiratory system material was negative, however, the analysis of the cerebrospinal fluid gave a positive result. There were no signs of cerebral edema, hyper intensive areas were observed in the temporal lobe, lateral ventricle, and hippocampus. CT scan revealed a picture characteristic of pneumonia. Pharmacotherapy consisting of antibiotics, steroids, and antiviral drugs such as acyclovir and favipiravir was implemented [159]. Paterson et al. [160] observed that SARS-CoV-2 infection is associated with a wide spectrum of neurological syndromes affecting the whole neuraxis, including the cerebral vasculature and, in some cases, responding to immunotherapy. The high incidence of acute disseminated encephalomyelitis, particularly with haemorrhagic changes, is demonstrated. The infection of the CNS is most likely due to the entry of olfactory pathways [79].

### 7.1. SARS-CoV-2—Children

Song et al. [131] described the transmission of SARS-CoV-2 in four families. Each family had one index case, which had direct or indirect contact with a person from Wuhan. In 22 out of 24 family members, the SARS-CoV-2 RNA presence was confirmed. The disease manifestations, latent period, and virus shedding period varied a lot. Amongst the 22 infected family members, 20 (90.1%) had mild symptoms and only two (8.9%) had severe clinical manifestations. A prolonged period of virus shedding in the upper respiratory tract after symptom resolution was observed in 25.0% of cases (these patients were still SARS-CoV-2 RNA positive).

Data on SARS-CoV-2 infection in children are limited [161]. The first case of SARS-CoV-2 infection in children was reported in Shenzhen on 01/20/2020 [162]. Sun D et al. [161] reported the infection in children as a result of recent direct contact with infected relatives, usually grandparents. Among the children included in the study, 51.4% of children were diagnosed with pneumonia (CT), whereas 23.0% remained asymptomatic carriers of the virus, being a source of SARS-CoV-2 transmission [161]. None of the studied children with COVID-19 had lymphopenia, which occurs in adults. The median age of infected children was 5.8 years with no notable gender difference [134]. Wei et al. [163] in their review showed that between 9 December 2019 and 6 February 2020, there were nine infants diagnosed with COVID-19 in China. The youngest patient was one month old, and the oldest was 11 months old. Infants infected with SARS-CoV-2 showed variable clinical symptoms, four patients had fever, two mild respiratory symptoms, and one patient showed no symptoms of the disease. Both studies mentioned above revealed the significant finding that families of infected children and infants had at least one family member diagnosed with COVID-19. Data on pediatric COVID-19 cases were collected also by Götzinger et al. [164]. Five hundred eighty-two children with SARS-CoV-2 infection, with a median age of 5.0 years, were included in the study. Twenty-five percent of the examined children had pre-existing disease, and four children died.

Children are less susceptible to SARS-CoV-2 infection and have longer incubation and fecal viral excretion time [165,166]. Overall, most infected infants and children have mild clinical signs and good prognosis. However, some of the pediatric patients require hospitalization. The duration of COVID-19 is approximately 1–2 weeks from the onset of the disease [163,167,168].

### 7.2. Possibility of Transmission from Mother to Child—Meta-Analysis

The purpose of this part study is to systematically accumulate current knowledge in the field and evaluate the possibility of transmission of SARS-CoV-2 from mother to child during childbirth.

#### 7.2.1. Methods

A systematic search was performed via databases of PubMed, Web of Science, and Google Scholar (intitle) up to 16 January 2021. The Medical Subject Headings (MeSH) terms will be: (gravidity OR gestation OR pregnancy OR pregnant women OR labour OR preterm labour OR obstetric OR maternal-fetal exchange) OR (fetal OR neonate OR newborn OR vaginal delivery OR cesarean delivery OR neonatal morbidity OR neonatal mortality) AND (coronavirus disease 2019 OR severe acute respiratory syndrome coronavirus 2 OR SARS-CoV-2 infection OR 2019-nCoV disease OR COVID-19 virus disease OR COVID-19 virus infection). Two independent researchers (KG-B and NW-K) screened eligible articles that met the analysis’s inclusion and exclusion criteria. The inclusion criteria were (1) information about infected mothers, (2) information about infected newborns, and (3) information about the type of childbirth. There was a language limitation in this search (only English articles). We excluded review articles, metanalysis, guidelines, recommendations, case reports, and letters to the editor.

All the analyses were performed at a significance level α = 0.05. The meta-analysis was performed with the fixed effects model to compare the odds ratio of neonates infection in vaginal birth and caesarean birth. For the heterogeneity analysis of the studies, Cochran’s Q statistics were applied. The estimator T2 and I2 statistics were also computed. The main results of the meta-analysis were showed as the forest plot of OR with 95% confidence intervals. The publication bias assessment was analyzed using Begg and Mazumdar test.

#### 7.2.2. Results and Limitations

**Figure 4:**
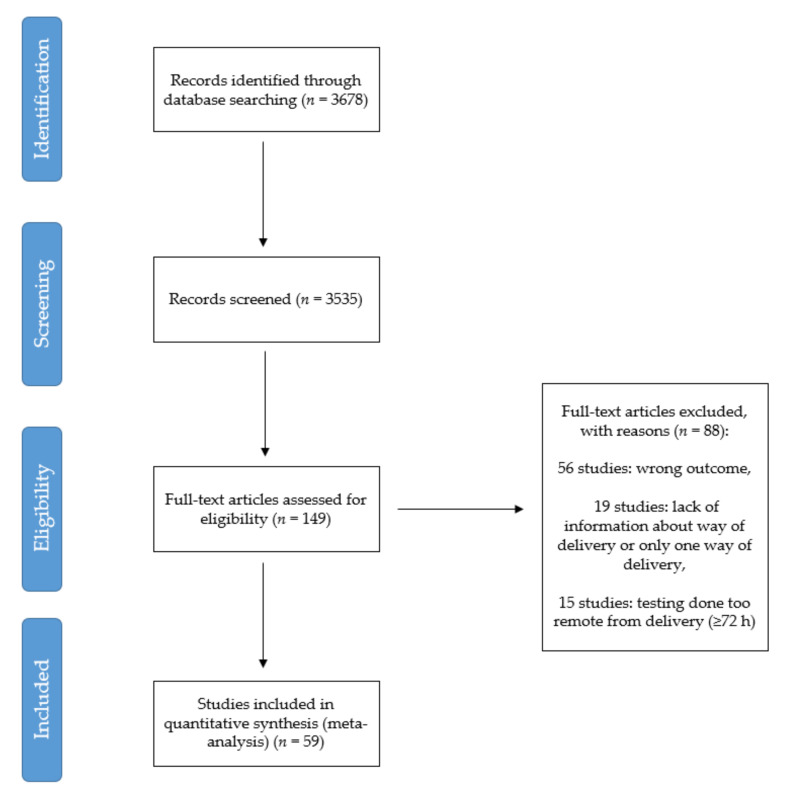
Search plot diagram.

Figure 4 schematically illustrates the outcomes of the literature search. Of 3678 articles identified, 149 full manuscripts were assessed further for eligibility, and 59 studies were included (Supplementary Materials Table S1).

**Figure 5:**
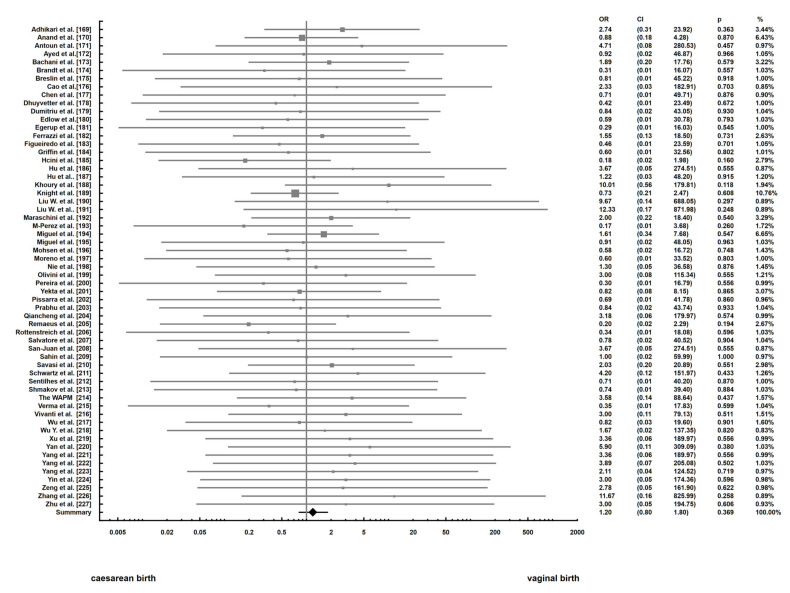
Forest plots estimating the odd ratios of infection in vaginal birth and caesarean birth [169,170,171,172,173,174,175,176,177,178,179,180,181,182,183,184,185,186,187,188,189,190,191,192,193,194,195,196,197,198,199,200,201,202,203,204,205,206,207,208,209,210,211,212,213,214,215,216,217,218,219,220,221,222,223,224,225,226,227].

The analysis was focused on the comparisons of OR of neonates’ infection in vaginal birth and caesarean birth. The forest plot presents results of the meta-analysis (Figure 5). The main finding from our study is that there is no statistically significant difference between odds ratio of neonates’ infection in vaginal birth and caesarean birth (*p* = 0.369). The heterogeneity analysis showed (Q = 21.9, T^2^ = 0, I^2^ = 0, *p* = 1) that we cannot reject the hypothesis that the variance in effect size is produced only by sampling error.

In the research based on a meta-analysis, one should pay special attention to the publication bias. We conducted a Trim-and-Fill analysis for describing it and constructed a funnel plot. The plot shows that the distribution of the effect size is symmetrical. We also performed Begg and Mazumdar rank correlation test to examine the association between effect estimates and their variances. The results of the test are as follows: τ=0.135, p=0.13. The meta-analysis was based only on papers published in English.

#### 7.2.3. Discussion and Conclusion

The transmission of the virus from mother to child is rare, and the infection rate is not higher in the case of natural childbirth, breastfeeding, or contact with the mother. Children who have SARS-CoV-2 infection are mostly asymptomatic [228,229,230]. Thus far, the transmission of the virus through the placenta of pregnant women has not been proven [2,7,8,16,77,120,231,232,233,234]. Penfield et al. [235] found the virus in 3 of the 11 placental samples tested, but no neonatal infection was detected during the first five days of life. The authors have not confirmed the vertical transmission of the virus from mother to child, which agrees with our results of the meta-analysis. Nevertheless, infection of SARS-CoV-2 during pregnancy may be a threat to the fetus due to the poor clinical condition of the mother and the possible occurrence of intrauterine growth disorders [236]. The patient with a positive result should be quarantined for a standard time or hospitalized in the case of a severe clinical condition. Postpartum women with diagnosed COVID-19 should undergo anticoagulation treatment [237], and the child’s well-being should be monitored by ultrasound and Doppler. A decision on delivery should include an individual situation, i.e., gestational age or clinical condition of the mother [238]. It is not entirely clear whether the virus can pass through breast milk. Most available works on this topic do not report on [239,240,241,242,243,244,245,246], a live virus in milk. Tam et al. [247] were the first to detect SARS-CoV-2 RNA in breast milk. The virus was detected in two independent milk samples of a woman with mild symptoms, taken 10 days apart. Similar results showed Groß et al. [248]. In both works, the authors emphasize that the detection of RNA does not indicate the presence of the live virus in breast milk and the mechanism of viral transmission via breast milk requires further investigation. It should be remembered that breast milk is the best source of nutrients for infants and provides protection against many diseases due to the presence of many immunoglobulins and bioactive ingredients. However, no studies unequivocally showing the presence of anti-SARS-CoV-2 antibodies in breast milk are available. For this reason, we approve WHO recommendations that SARS-CoV-2 positive mothers should continue breastfeeding or when their clinical condition is severe use expressed breastmilk following WHO precautions [249].

## 8. Diagnostics

An important element of the diagnosis is a correctly conducted interview, focused on patients’ contact with sick people and recent journeys. As COVID-19 in mild form may resemble the clinical course of flu or cold, a differential diagnosis is necessary [16,120]. Differential diagnostics should also be performed in the case of organizing pneumonia, pneumonia of bacterial, chlamydial, and mycoplasmic etiology, as well as dermatomyositis and vasculitis [43].

### 8.1. Medical Imaging Tests

A technique allowing confirmation of COVID-19 incidence is a CT (computed tomography) scan. The resulting image corresponds to pneumonia. In many cases, this examination seems to be decisive for excluding or confirming the diagnosis, but often no pathologies are found in patients. A characteristic element of COVID-19 is the ground-glass opacity image, occurring in approximately 56% to over 76% of patients. Bilateral patchy shadowing and segmental consolidation are also common [10,120]. Classical X-ray examination is also used in COVID-19 diagnostics, although it is less effective than a CT scan. At the initial stage of the disease, interstitial changes and numerous shadows of heterogeneous shape and small size appear [43]. Patients in the advanced clinical stage have lesions, shown on x-rays as lung consolidation with the possible presence of pleural effusion, infiltrating shadows, and ground-glass opacity characteristics for SARS-CoV-2 infection [43]. In children, the X-ray image may not show any abnormalities [250].

### 8.2. Laboratory Diagnostic Tests

**Figure 6:**
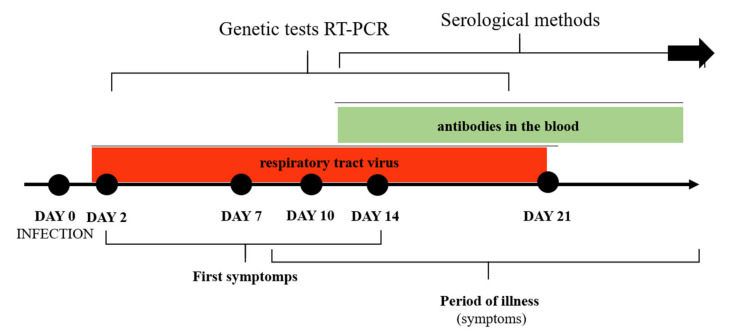
Stages of infection and possibility of using genetic and serological tests.

The American Society of Microbiology in a report of 23 March 2020, divides the tests into two main groups: based on the detection of SARS-CoV-2 genetic material and the immune response after contact with virus particles. The first group allows confirmation of active COVID-19 cases, which is particularly important for limiting the spread of the virus. The second group serves for epidemiological or screening tests [251] (Figure 6).

#### 8.2.1. Molecular Tests and Their Implications

Currently, the “gold standard” for SARS-CoV-2 detection worldwide is RT-PCR [252]. According to the references of the World Health Organization (WHO), depending on the test used, 2 or 3 genes common to the Sarbecovirus subgenus (E gene) and genes characteristic only for SARS-CoV-2 (N, RdRp or ORF1ab) are identified. However, the disadvantage of this method is a relatively high probability of a false negative result, reaching about 30–50% (sensitivity ranges from 50–79%) [70,120,253,254,255]. Currently, many commercial tests, including RNA, are available. On 18 March 2021, the FIND organization listed 391 molecular assays available on the market and approved for in vitro diagnostic (www.finddx.org/COVID-19/pipeline, accessed on 18 March 2021) by US FDA EUA (71 tests) or/and with CE-IVD certificate (255 tests). Some kits allow RNA isolation and PCR in one panel, which significantly shortens the time of sample preparation. Wozniak et al. [256] also described the non-commercial possibilities of viral RNA extraction based on basic reagents available in each laboratory.

During analyzing the RT-PCR results, the possibility of obtaining a false negative result in the early stages of infection should be taken into account. Molecular tests show maximum sensitivity only after about 7 days from the human contact with the virus. After a dozen days of the first disease symptoms, the sensitivity of genetic tests gradually declines, which may result in a false-negative result. This is due to a decrease in the number of virus particles in the respiratory epithelium [251,257,258,259,260].

To diagnose COVID-19 disease, it is possible to take nasopharyngeal, or mid-turbinate or nasal swabs as well as to analyze the material derived from bronchoalveolar lavage, saliva, tears, feces, urine, or sputum [2,44,123,162,257,258,259,260]. There are cases in which tests from materials from the respiratory tract were SARS-CoV-2 negative, whereas tests from the rectal material gave positive results [251]. Similarly, the material taken from the upper section of the respiratory tract could be SARS-CoV-2 negative and the lower section positive [251].

Data comparing the accuracy of RT-PCR testing suggest that the test sensitivity may vary by type of specimen. The most accurate sample for the diagnosis of COVID-19 was sputum, followed by nasal or nasopharyngeal swabs [261]. As viral particles exhibit “enhanced binding” to the olfactory epithelium, Butowt et al. [79] underline the importance of the upper respiratory tract material. Czumbel et al. [262] in meta-analysis indicated that saliva is a candidate for COVID-19 diagnostic tests. They found 91% (95%CI = 80–99%) sensitivity for saliva tests and 98% (95%CI 89–100%) sensitivity for nasopharyngeal swab tests. In most comparative studies, drooled saliva had higher positivity rates than deep saliva/sputum. The positivity of saliva was 31–92% depending on the cohort and length of hospitalization. Saliva is not recommended by WHO for routine clinical diagnostics of COVID-19 [263], but received FDA Emergency Use Authorization (FDA-EUA). Actually, there are 5 tests based on saliva with FDA authorization. This specimen is easier to collect than other materials and could be potentially used in self-monitoring by healthcare workers or be used in the research of large groups of people, i.e., in medicine students before clinical tutorials starting [264].

The positive result of the RT-PCR reaction may represent either viral residues or infectious virus particles. Copies of RNA obtained by using RT-qPCR method in a specimen are the sum of non-infectious and infectious RNA per 1 mL. It is important to know the relationship between genomic copies per ml and plaque-forming unit [PFU] that is specific only for infectious viral particles. Houng et al. [252], using the best-fit dose–response model, showed that 1 PFU corresponds to 1239 copies of SARS-CoV-1. If we adopt this correlation for SARS-CoV-2, we can predict that even 10^3^ copies of SARS-CoV-2 RNA are able to start infection.

Wölfel et al. [265] found a high level of viral RNA in stool samples of nine examined patients with confirmed COVID-19 even over three weeks after the decline of symptoms, but infectious viruses were not found. A similar situation was observed for specimens collected from sputum and nasopharyngeal swabs of patients a few days after the resolution of symptoms [265]. Therefore, RNA tests should not be used in patients after the decline of disease symptoms to confirm convalescence [183]. It has been proposed to use the test based on viral sub-genomic mRNA, that is transcribed only in infected cells and is not packaged into virions. The presence of this form of RNA might indicate the presence of active viruses in the patient [265].

The peak concentration of SARS-CoV-2 RNA in the specimen is 1000 times higher than for SARS-CoV-1 and MERS-CoV. This peak for SARS-CoV-2 is reached 2–5 days earlier than for the rest of CoVs [265]. The high concentration of SARS-CoV-2 virus particles in sputum and probably low infectious dose might be related to the higher spreading ability of SARS-CoV-2 in comparison with MERS and SARS-CoV-1. Along with the decline of symptoms, RNA concentration decreases and after ten days of symptom onset is less than 10^5^ viral RNA copies per 1 mL of sputum. Wölfel et al. [265] did not isolate infectious viruses from samples containing less than 10^6^ viral RNA copies per ml. This finding disagrees with the Houng et al. model [252] and may suggest that infected patients after ten days of symptom decline are non-infectious for other people.

#### 8.2.2. Serological Tests and Their Implications

**Figure 7:**
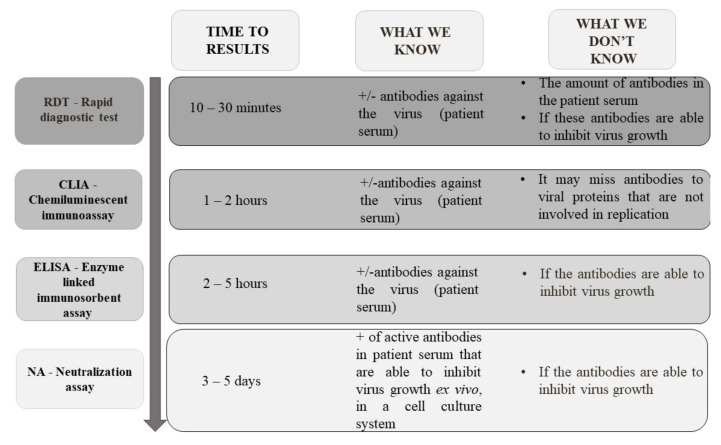
Types of serological tests based on antibodies used in SARS-CoV-2 diagnostics [263].

The next group of tests are serological tests, which allow the detection of antibodies in the patient’s serum, or blood or antigens in specimens from the respiratory tract. This group includes rapid diagnostic test (RDT), chemiluminescent immunoassay (CIA), enzyme-linked immunosorbent assay (ELISA), and neutralization assay (Figure 7) [43,70,79].

Antigen tests detect viral proteins expressed by the COVID-19 virus in a sample collected from the respiratory tract. If the sufficient concentration of antigen is present in the examined sample, a detectable line appears on the cassette test. This type of test is useful only if the concentration of the SARS-CoV-2 antigen is high, so it should be used only in acute or early stage of infection [266]. According to the last WHO recommendation [263], antigen-based tests could be implemented in the COVID-19 diagnostic algorithm if the performance of the test is acceptable. It is possible if the prevalence of SARS-CoV-2 in the examined population is high, and the sensitivity (≥80%) and specificity (≥97%) of RDT are sufficient. Actually, amongst the 147 commercialized tests with CE-IVD authorization, only 8 have US FDA-EUA approval (https://www.finddx.org/covid-19/pipeline/, accessed on 18 March 2021).

On 18 March 2021, the FIND organization listed 530 serological assays available on the market (www.finddx.org/covid-19/pipeline, accessed on 18 March 2021) with regulatory CE-IVD and/or US FDA EUA. The vast majority (380; 71.7%) are the so-called rapid “cassette” tests based on lateral-flow (LFIA) immunochromatography (Figure 7). The principle of these tests is based on the reaction of serum, plasma, or whole blood antibodies present in the sample with labeled, e.g., gold particles, virus antigens. The resulting complex migrates through the nitrocellulose membrane under the action of capillary forces and is eventually captured by the membrane-attached human immunoglobulin antibodies forming a red line. The advantage of these tests is the simplicity of implementation (no additional reagents or equipment to read the results are needed) and a very short time to obtain the result (usually 10–30 min). Nevertheless, this is not a quantitative method and does not allow determining whether serum antibodies inhibit the growth of the virus [267].

Chemiluminescence (CLIA) is another analytical method for SARS-CoV-2 antibodies detection. In this immunoassay, the reaction indicator may be a phosphor or enzyme marker. The method is characterized by a higher sensitivity compared to the RDT methods. The result is usually obtained within 2 h.

The level of specific IgA, IgG, and IgM antibodies in the tested serum samples is usually determined by ELISA immunoassay kits. Compared to RDT, ELISA tests generally have a much higher sensitivity and the ability to simultaneously detect antibodies in a large number of clinical samples. Table 2 summarizes all available serological tests on the day 18 March 2021 (*n* = 125) with US FDA EUA or/and with CE-IVD certification, excluding RDT tests, due to their low usefulness in diagnostics.

Combined immunochromatographic tests, based on the detection of specific IgG and IgM, are also used. These assays are fast (about 15 min) and can be performed regardless of the clinical advancement of the patients and the type of blood sample. The study on a small group of patients (397) revealed high sensitivity (almost 89%) and specificity (over 90%) of these tests [255]. Zhao et al. [268] have conducted serological tests on a group of 173 patients with diagnosed COVID-19. They analyzed over 500 plasma samples and then found the phenomenon of seroconversion in response to coronavirus infection. It occurred in approximately 93% (median of 11 days), 83% (median of 12 days), and in 65% of the samples (median of 14 days), in the case of IgA IgM, and IgG, respectively. From the 15th day of infection, the level of all 3 classes immunoglobulins increased, while the diagnostic capabilities of the SARS-CoV-2 genetic material-based methods decreased [268].

To date, it has not been established whether the infection gives immunity to COVID-19, as well as if the immunity is total or partial, and how long it lasts. The authors point out that based on tests on an animal model, it can be assumed that the recovery most likely contributes to the development of protective immunity [251]. Investigation showed that people without SARS infection history who worked on “wet markets” in Southern China, with animals being a reservoir of SARS-CoV-1, had antibodies against SARS-CoV-1 [135]. Other market employers who did not have contact with those mammals (such as Himalayan palm civets, raccoon dogs) had no antibodies against SARS-CoV-1. Unfortunately, the humoral response in patients after SARS-CoV-1 infection is short [269]. Antibodies of IgG class against SARS-CoV-1 were reduced about 3 years after infection [270]. All 176 patients with SARS-CoV-1 history had detectable levels of IgG after 90 days of symptom onset. This level decreased after a further 130 days [270]. Antibodies against MERS-CoV, including neutralizing antibodies, were detectable in 6 out of 7 examined patients until 34 months after symptomatic (various stages) infection [271]. However, antibody titers in patients with a mild form of MERS-CoV infection confirmed by using the RT-PCR method were below the detectable level 12 months after infection [272]. In some groups of patients with MERS-CoV infection, antibodies were not detected at all [273]. A serological analysis in 12 MERS-CoV infected patients revealed that early humoral response reduced the disease severity. Decreased level of neutralizing antibodies against MERS-CoV occurred about 20 days after illness onset [273]. In SARS-CoV-2 neutralizing antibody pick is observed earlier, 10–15 days after the disease onset. It may explain the lower mortality of COVID-19 in comparison with MERS-CoV disease. Robbiani et al. [274] observed that the level of neutralizing activity of antibodies in the plasma of patients with confirmed SARS-CoV-2 infection is low after 39 days of illness onset.

Observation of anti-SARS-CoV-2 antibody dynamics is very helpful in predicting COVID-19 epidemiology and the effectiveness of possible vaccination. Serological tests allow the determination of the real spread of SARS-CoV-2 in different populations. According to the last WHO recommendations, the detection of anti-SARS-CoV-2 antibodies could be used for the confirmation COVID-19 case in patients with symptoms and with negative NAAT results for SARS-CoV-2 [262].

Comparative analysis of the various available laboratory methods is very needed. Only the comparison with the currently recommended “gold standard” methods (virus culture or NAAT), allows choosing the most specific and sensitive methods. This in turn, may help to extend diagnostics in most sites of the world and expand our knowledge about the prevalence of COVID-19 pandemic.

#### 8.2.3. Other Methods

Song et al. [275] identified high-binding-affinity aptamers targeting SARS-CoV-2 RBD, using an ACE2 competition-based aptamer selection strategy and a machine learning screening algorithm. These aptamers present an opportunity for generating new probes for the recognition of SARS-CoV-2 and could assist in the diagnosis and treatment of SARS-CoV-2. Based on laboratory testing, COVID-19 should be suspected in the case of normal or elevated monocyte levels, normal or reduced leukocyte levels, and simultaneous lymphocyte deficiency [43]. There is also an increased concentration of inflammatory indicators: CRP, procalcitonin, D-Dimers, and amyloid A serum [276]. SARS-CoV-2 may also damage thrombocytes, inhibit their production and release from myeloid megakaryocytes, leading to thrombocytopenia. Such a condition is associated with higher mortality. The risk of death is inversely proportional to the level of thrombocytes [277,278,279,280].

## 9. Summary Points

1. Transmission of the SARS-CoV-2 virus from animals to humans cannot be excluded.
2. Abiotic surfaces are also a source of nosocomial infections and direct contact with contaminated surfaces in public can contribute to the spread of SARS-CoV-2.
3. Transmission of SARS-CoV-2 through the placenta has not yet been confirmed. The performed meta-analysis showed no relationship between the type of delivery and the frequency of infections in newborns of mothers infected with SARS-CoV-2. The environment and saliva might play an important role in virus transmission.
4. The presence of viral RNA is not an equivalent of active viral infection.
5. Tests based on viral, sub-genomic mRNA or serological methods should be used to confirm the convalescence.
6. It is important to find the relationship between genomic copies per ml of various specimens and plaque-forming units to predict the number of SARS-CoV-2 RNA copies able to start infection. It could help in describing all potential transmission routes.
7. The high concentration of SARS-CoV-2 particles in human specimens and probably low infectious dose might be related to the high spreading ability of COVID-19.
8. The wide comparative analysis of various commonly available laboratory methods with gold standards in the SARS-CoV-2 diagnostic method (virus culture or NAAT) is needed to extend the knowledge about the COVID-19 pandemic.
9. Quarantine seems to be the best way to overcome the pandemic of COVID-19.

## Figures and Tables

**Table 1:** Clinical manifestations of COVID-19 infection [7,142].

Mild COVID-19	Moderate COVID-19	Severe COVID-19
⮚ Low fever (up to 38 degrees) ⮚ Nasal congestion ⮚ Dry cough ⮚ Muscle pain ⮚ Headache ⮚ Sore throat ⮚ Shortened breath ⮚ No clinical features of pneumonia ⮚ Diarrhea	⮚ Moderate form of pneumonia ⮚ Cough ⮚ Breathlessness ⮚ Tachypnea—in the pediatric population	⮚ Severe form of pneumonia ⮚ High or low fever ⮚ Advanced dyspnea ⮚ Tachypnea ⮚ Saturation below 90%, as a feature of body hypoxia (according to other sources 93%) cyanosis—in the pediatric population ⮚ ARDS ⮚ Hypoxaemia ⮚ Organ dysfunction ⮚ Sepsis

**Table 2:** A list of selected serological tests on regulatory of CE-IVD and/or US-FDA EUA for the detection of antibodies against the SARS-CoV-2 virus.

Test Target	Test Type	Class of Antibody	Number of Commercialized Test
**Antibody**	Antibody Microarray	IgM, IgG	2
	Immunofluorescent assay	IgM, IgG	2
	EIA	IgM, IgG and IgA	6
	ELISA	IgA	12
		IgG	54
		IgM	20
		IgM, IgA	3
		IgM, IgG	6
		IgM, IgG, and IgA	9
	CLIA	IgG	6
		IgM	2
		IgM, IgA	1
		IgM, IgG, and IgA	2
	CMIA	IgG	1
	ECLIA	IgG, IgM	1
	Up-converting Phosphor Immunochromatografic Technology	IgG, IgM	1

EIA—Enzyme Immunoassay; ELISA—Enzyme Linked Immunosorbent Assays; CLIA—Chemiluminescence Immunoassay; CMIA—Chemiluminescent Microparticle Immunoassay; CE-IVD—European CE Marking for In Vitro Diagnostic; US FDA EUA—U.S. Food and Drug Administration Emergency Use Authorization.

## Data Availability

Not applicable.

## Supplementary Materials

The following are available online at https://www.mdpi.com/article/10.3390/jcm10091962/s1, Table S1: Characteristics of included studies in meta-analysis.
