# Supplementary material for: SARS-CoV-2—Morphology, Transmission and Diagnosis during Pandemic, Review with Element of Meta-Analysis
Source: J Clin Med. 2021 May 3;10(9):1962. doi: 10.3390/jcm10091962 (PMC8125301; doi:10.3390/jcm10091962)
Supplement: Supplementary file 1 [file jcm-10-01962-s001.zip › jcm-1180318-supplementary.pdf]

Number of study	Title	Authors	Country	N of woman with SARS-CoV-2	Total neonates vaginal birth	Infected neonates vaginal birth	Total neonates caesarean birth	Infected neonates caesarean birth	Author's conclusion	Ref.
1	Pregnancy Outcomes Among Women With and Without Severe Acute Respiratory Syndrome Coronavirus 2 Infection	Adhikari et al.	USA	188	123	5	65	1	Neonatal infection may be as high as 3% and may occur predominantly among asymptomatic or mildly symptomatic women.	169
2	Clinical profile, viral load, management and outcome of neonates born to COVID 19 positive mothers: a tertiary care centre experience from India	Anand et al.	India	65	39	4	26	3	Risk of transmission from COVID 19 positive mother to neonate by rooming-in and breastfeeding is low.	170
3	Maternal COVID-19 infection, clinical characteristics, pregnancy, and neonatal outcome:	Antoun et al.	UK	19	3	0	16	0	COVID-19 infection was not found in the newborns and	171

Number of study	Title	Authors	Country	N of woman with SARS-CoV-2	Total neonates vaginal birth	Infected neonates vaginal birth	Total neonates caesarean birth	Infected neonates caesarean birth	Author's conclusion	Ref.
	A prospective cohort study								none developed severe neonatal complications.	
4	Maternal and perinatal characteristics and outcomes of pregnancies complicated with COVID-19 in Kuwait	Ayed et al.	Kuwait	165	86	0	79	0	Mother-to-child vertical transmission of SARS-CoV-2 is possible, COVID-19 infection during pregnancy may not lead to unfavorable maternal and neonatal outcomes.	172
5	Clinical Profile, Viral Load, Maternal-Fetal Outcomes of Pregnancy With COVID-19: 4-Week Retrospective, Tertiary Care Single-Centre	Bachani et al.	India	83	57	4	26	1	The potential mother-to-child vertical/horizontal transmission needs further study.	173

Number of study	Title	Authors	Country	N of woman with SARS-CoV-2	Total neonates vaginal birth	Infected neonates vaginal birth	Total neonates caesarean birth	Infected neonates caesarean birth	Author's conclusion	Ref.
	Descriptive Study									
6	Epidemiology of coronavirus disease 2019 in pregnancy: risk factors and associations with adverse maternal and neonatal outcomes	Brandt et al.	USA	61	47	0	14	0	Coronavirus disease 2019 during pregnancy is associated with an increased risk of adverse maternal and neonatal outcomes	174
7	Coronavirus disease 2019 infection among asymptomatic and symptomatic pregnant women: two weeks of confirmed presentations to an affiliated pair of New York City hospitals	Breslin et al.	USA	18	10	0	8	0	There were no confirmed cases of coronavirus disease 2019 detected in neonates upon initial testing on the first day of life.	175
8	Clinical analysis of ten pregnant	Cao et al.	China	4	1	0	3	0	COVID-19 is not an	176

Number of study	Title	Authors	Country	N of woman with SARS-CoV-2	Total neonates vaginal birth	Infected neonates vaginal birth	Total neonates caesarean birth	Infected neonates caesarean birth	Author's conclusion	Ref.
	women with COVID-19 in Wuhan, China: A retrospective study								indication of cesarean section.	
9	Clinical analysis of pregnant women with 2019 novel coronavirus pneumonia	Chen et al.	China	5	3	0	2	0	No complications were observed in the newborn. Pregnancy and perinatal outcomes of patients with COVID-19 should receive more attention.	177
10	Coronavirus Disease 2019 in Pregnancy: The Experience at an Urban Safety Net Hospital	Dhuyvetter et al.	USA	21	15	0	6	0	Despite these risk factors, the patients uniformly had either mild or asymptomatic disease.	178
11	Outcomes of Neonates Born to Mothers With Severe Acute Respiratory	Dumitriu et al.	USA	101	55	0	46	0	No clinical evidence of vertical transmission was identified	179

Number of study	Title	Authors	Country	N of woman with SARS-CoV-2	Total neonates vaginal birth	Infected neonates vaginal birth	Total neonates caesarean birth	Infected neonates caesarean birth	Author's conclusion	Ref.
	Syndrome Coronavirus 2 Infection at a Large Medical Center in New York City								in newborns of mothers positive for SARS-CoV-2 or with suspected infection.	
12	Assessment of Maternal and Neonatal SARS-CoV-2 Viral Load, Transplacental Antibody Transfer, and Placental Pathology in Pregnancies During the COVID-19 Pandemic	Edlow et al.	Israel	57	36	0	21	0	The study indicated no evidence of placental infection or definitive vertical transmission of SARS-CoV-2.	180
13	Severe Acute Respiratory Syndrome Coronavirus 2 (SARS-CoV-2) Antibodies at Delivery inWomen, Partners, and Newborns	Egerup et al.	Denmark	28	22	0	6	0	In this study authors found no association between SARS-CoV-2 infection and obstetric or neonatal complications.	181

Number of study	Title	Authors	Country	N of woman with SARS-CoV-2	Total neonates vaginal birth	Infected neonates vaginal birth	Total neonates caesarean birth	Infected neonates caesarean birth	Author's conclusion	Ref.
14	Vaginal delivery in SARS-CoV-2-infected pregnant women in Northern Italy: a retrospective analysis	Ferrazzi et al.	Italy	42	24	2	18	1	The report showed that vaginal delivery is associated with a low risk of intrapartum SARS-Cov-2 transmission to the newborn.	182
15	Systematic screening for SARS-CoV-2 in pregnant women admitted for delivery in a Portuguese maternity	Figueiredo et al.	Portugal	184	126	0	58	0	Systematic screening for SARS-CoV-2 is very important aspect because of asymptomatic infection.	183
16	The Impact of COVID-19 Infection on Labor and Delivery, Newborn Nursery, and Neonatal Intensive Care Unit: Prospective Observational	Griffin et al.	USA	27	17	0	10	0	No infants had clinical evidence of symptomatic COVID-19 infection, but 14 of them were positive for SARS-CoV-2.	184

Number of study	Title	Authors	Country	N of woman with SARS-CoV-2	Total neonates vaginal birth	Infected neonates vaginal birth	Total neonates caesarean birth	Infected neonates caesarean birth	Author's conclusion	Ref.
	Data from a Single Hospital System									
17	Maternal, fetal and neonatal outcomes of large series of SARS-CoV-2 positive pregnancies in peripartum period: A single-center prospective comparative study	Hcini et al.		108	101	3	7	1	Neonate from positive SARS-CoV-2 mothers had no outcomes and were SARS-CoV-2 negative	185
18	Managing Preterm Infants Born to COVID-19 Mothers: Evidence from a Retrospective Cohort Study in Wuhan, China	Hu et al.	China	6	1	0	5	0	The risk of vertical transmission of SARS-CoV-2 is low in preterm infants born to COVID-19 mothers if appropriate management is implemented.	186
19	Severe Acute Respiratory Syndrome Coronavirus 2	Hu et al.	China	7	1	0	6	1	The findings suggest that the vertical transmission of	187

Number of study	Title	Authors	Country	N of woman with SARS-CoV-2	Total neonates vaginal birth	Infected neonates vaginal birth	Total neonates caesarean birth	Infected neonates caesarean birth	Author's conclusion	Ref.
	(SARS-CoV-2) Vertical Transmission in Neonates Born to Mothers With Coronavirus Disease 2019 (COVID-19) Pneumonia								SARS-CoV-2 infection from mothers affected by COVID-19 during the last days of pregnancy is possible but relatively infrequent.	
20	Characteristics and Outcomes of 241 Births to Women With Severe Acute Respiratory Syndrome Coronavirus 2 (SARS-CoV-2) Infection at Five New York City Medical Centers	Khoury et al.	USA	236	136	6	100	0	COVID-19 severity was associated with higher rates of cesarean and preterm birth, but nearly all newborns tested were negative for SARS-CoV-2 immediately after birth.	188
21	Characteristics and outcomes of pregnant women admitted to	Knight et al.	UK	262	106	4	156	8	Most pregnant women had good outcomes, and	189

Number of study	Title	Authors	Country	N of woman with SARS-CoV-2	Total neonates vaginal birth	Infected neonates vaginal birth	Total neonates caesarean birth	Infected neonates caesarean birth	Author's conclusion	Ref.
	hospital with confirmed SARS-CoV-2 infection in UK: national population based cohort study								transmission of SARS-CoV-2 to infants was uncommon.	
22	Clinical Analysis of Neonates Born to Mothers with or without COVID-19: A Retrospective Analysis of 48 Cases fromTwo Neonatal Intensive Care Units in Hubei Province	Liu W. et al.	China	15	1	0	14	0	No evidence of vertical transmission was found in the study.	190
23	Clinical characteristics of 19 neonates born to mothers with COVID-19	Liu W. et al..	China	19	1	0	18	0	In this study no vertical transmission of SARS-CoV-2 and no perinatal complications in the third trimester was found .	191

Number of study	Title	Authors	Country	N of woman with SARS-CoV-2	Total neonates vaginal birth	Infected neonates vaginal birth	Total neonates caesarean birth	Infected neonates caesarean birth	Author's conclusion	Ref.
24	Coronavirus and birth in Italy: results of a national population-based cohort study	Maraschini et al.	Italy	146	98	4	48	1	Clinical features and outcomes of COVID-19 in women and their infants are similar to those described for the general population.	192
25	Association Between Mode of Delivery Among Pregnant Women With COVID-19 and Maternal and Neonatal Outcomes in Spain	Martínez-Perez et al.	Spain	78	41	0	37	2	None newborns developed COVID-19 symptoms within 10 days after birth, all tested samples were negative.	193
26	Maternal, Perinatal and Neonatal Outcomes With COVID-19: A Multicenter Study of 242 Pregnancies and Their 248 Infant Newborns During	Marín Gabriel et al.	Spain	242	179	9	63	2	In this study no COVID-19 transmission during delivery or throughout the first month of life in the newborns was detected.	194

Number of study	Title	Authors	Country	N of woman with SARS-CoV-2	Total neonates vaginal birth	Infected neonates vaginal birth	Total neonates caesarean birth	Infected neonates caesarean birth	Author's conclusion	Ref.
	Their First Month of Life									
27	Multicentre Spanish study found no incidences of viral transmission in infants born to mothers with COVID-19	Marín Gabriel et al.	Spain	42	22	0	20	0	There was no evidence of COVID-19 transmission in any of the infants born to COVID-19 mothers.	195
28	Characteristics of Newborns Born to SARS-CoV-2-Positive Mothers: A Retrospective Cohort Study	Mohsen et al.	USA	15	5	0	10	1	Neonates born to mothers with confirmed or suspected SARS-CoV-2 are most of the time asymptomatic. Vertical transmission should be studied thoroughly.	196
29	Vertical Transmission of COVID-19 to the Neonate	Moreno et al.	USA	19	12	0	7	0	In this report, symptomatic COVID-19 during the	197

Number of study	Title	Authors	Country	N of woman with SARS-CoV-2	Total neonates vaginal birth	Infected neonates vaginal birth	Total neonates caesarean birth	Infected neonates caesarean birth	Author's conclusion	Ref.
									pregnancy was not associated with vertical transmission to the neonate.	
30	Clinical features and the maternal and neonatal outcomes of pregnant women with coronavirus disease 2019	Nie et al.	China	27	5	0	22	1	The risk of perinatal transmission of SARS-CoV-2 was low. Only 1 out of 26 newborns had confirmed SARS-CoV-2 infection.	198
31	A neonatal cluster of novel coronavirus disease 2019: clinical management and considerations	Olivini et al.	Italy	5	2	2	3	2	This study indicated that affected neonates were asymptomatic or paucisymptomatic. Despite these reassuring findings, a few cases of severe presentation in	199

Number of study	Title	Authors	Country	N of woman with SARS-CoV-2	Total neonates vaginal birth	Infected neonates vaginal birth	Total neonates caesarean birth	Infected neonates caesarean birth	Author's conclusion	Ref.
									the neonatal population have been reported.	
23	A multicenter study on epidemiological and clinical characteristics of 125 newborns born to women infected with COVID-19 by Turkish Neonatal Society	Oncel et al.	Turkey	125	36	1	89	3	COVID-19 in pregnant women has important impact on perinatal and neonatal outcomes.	200
33	Clinical course of Coronavirus Disease-2019 (COVID-19) in pregnancy	Pereira et al..	Spain	23	18	0	5	0	No vertical or horizontal transmissions were diagnosed in the neonates during labor or breastfeeding.	201
34	Perinatal management of SARS-CoV-2 infection in a level III	Pissarra et al.	Portugal	10	6	0	4	0	No newborn developed clinical signs of infection and no evidence of vertical	202

Number of study	Title	Authors	Country	N of woman with SARS-CoV-2	Total neonates vaginal birth	Infected neonates vaginal birth	Total neonates caesarean birth	Infected neonates caesarean birth	Author's conclusion	Ref.
	University Hospital								transmission was found.	
35	Pregnancy and postpartum outcomes in a universally tested population for SARS-CoV-2 in New York City: a prospective cohort study	Prabhu et al.	USA	70	38	0	32	0	The authors observed increased caesarean delivery rates and increased frequency of maternal complications in the postpartum period among pregnant women with COVID-19.	203
36	Coronavirus disease 2019 in pregnancy	Qiancheng et al.	China	22	5	0	17	0	No evidence supported vertical transmission of COVID-19 in the late stage of pregnancy, including	204

Number of study	Title	Authors	Country	N of woman with SARS-CoV-2	Total neonates vaginal birth	Infected neonates vaginal birth	Total neonates caesarean birth	Infected neonates caesarean birth	Author's conclusion	Ref.
									vaginal delivery.	
37	Characteristics and short-term obstetric outcomes in a case series of 67 women tested positive for SARS-CoV-2 in Stockholm, Sweden	Remaeus et al.	Sweden	67	47	1	20	2	Sixty-seven test-positive women delivered SARS-CoV-2-negative healthy neonates by vaginal birth.	205
38	Vaginal delivery in SARS-CoV-2-infected pregnant women in Israel: a multicenter prospective analysis	Rottenstreich et al.	Israel	52	39	0	13	0	In this prospective study among SARS-CoV-2-infected mothers, all neonatal was negative, and none of the infants developed pneumonia.	206
39	Neonatal management and outcomes during the COVID-19	Salvatore et al.	USA	82	46	0	36	0	This data suggest that perinatal transmission of	207

Number of study	Title	Authors	Country	N of woman with SARS-CoV-2	Total neonates vaginal birth	Infected neonates vaginal birth	Total neonates caesarean birth	Infected neonates caesarean birth	Author's conclusion	Ref.
	pandemic: an observation cohort study								COVID-19 is unlike because no neonates were positive for SARS-CoV-2.	
40	Incidence and clinical profiles of COVID-19 pneumonia in pregnant women: A single-centre cohort study from Spain	San-Juan et al.	Spain	6	1	0	5	0	Pregnant women with COVID-19 have a high risk of developing pneumonia. No cases of neonatal SARS-CoV-2 transmission was found.	208
41	A pandemic center's experience of managing pregnant women with COVID-19 infection in Turkey: A prospective cohort study	Sahin et al.	Turkey	10	5	0	5	0	The clinical course of COVID 19 during pregnancy appears to be mild in the present study.	209

Number of study	Title	Authors	Country	N of woman with SARS-CoV-2	Total neonates vaginal birth	Infected neonates vaginal birth	Total neonates caesarean birth	Infected neonates caesarean birth	Author's conclusion	Ref.
42	Clinical Findings and Disease Severity in Hospitalized Pregnant Women With Coronavirus Disease 2019 (COVID-19)	Savasi et al.	Italy	56	34	3	22	1	Preterm delivery occurred in 12% of patients, and nine newborns were admitted to the neonatal intensive care unit.	210
43	Chronic Histiocytic Intervillositis with Trophoblast Necrosis are Risk Factors Associated with Placental Infection from Coronavirus Disease 2019 (COVID-19) and Intrauterine Maternal-Fetal Severe Acute Respiratory Syndrome Coronavirus 2 (SARS-CoV-2)	Schwartz et al.		6	3	3	3	2	Potential mechanisms of infection of the placenta and fetus with SARS-CoV-2 are not confirmed.	211

Number of study	Title	Authors	Country	N of woman with SARS-CoV-2	Total neonates vaginal birth	Infected neonates vaginal birth	Total neonates caesarean birth	Infected neonates caesarean birth	Author's conclusion	Ref.
	Transmission in Liveborn and Stillborn Infants									
44	Coronavirus disease 2019 in pregnancy was associated with maternal morbidity and preterm birth	Sentilhes et al.	France	17	10	0	7	0	All neonates tested were negative for SARS-CoV-2.	212
45	Clinical course of novel COVID-19 infection in pregnant women	Shmakov et al.	Russia	40	23	0	17	0	No evidence of vertical transmission during pregnancy and delivery was found.	213
46	Maternal and Perinatal Outcomes of Pregnant Women with SARS-COV-2 infection	The WAPM (The World Association of Perinatal Medicine) working group on	22 different countries (Argentina, Australia, Belgium, Brazil, Colombi	251	115	1	136	0	The risk of vertical transmission seems to be negligible.	214

Number of study	Title	Authors	Country	N of woman with SARS-CoV-2	Total neonates vaginal birth	Infected neonates vaginal birth	Total neonates caesarean birth	Infected neonates caesarean birth	Author's conclusion	Ref.
		COVID-19	a, Czech Republic, Finland, Germany, Greece, Israel, Italy, North Macedonia, Peru, Portugal, Republic of Kosovo, Romania, Russia, Serbia, Slovenia, Spain, Turkey, and United States)							

Number of study	Title	Authors	Country	N of woman with SARS-CoV-2	Total neonates vaginal birth	Infected neonates vaginal birth	Total neonates caesarean birth	Infected neonates caesarean birth	Author's conclusion	Ref.
47	Outcomes of Maternal-Newborn Dyads After Maternal SARS-CoV-2	Verma et al.	USA	152	113	0	39	0	There was no distinct evidence of vertical transmission from mothers with SARS-CoV-2 to their newborns.	215
48	Retrospective Description of Pregnant Women Infected with Severe Acute Respiratory Syndrome Coronavirus 2, France	Vivanti et al.	France	33	17	1	16	0	In addition to maternal respiratory symptoms, neonatal conditions related to spontaneous or induced prematurity in relation to SARS-CoV-2 infection must be considered.	216
49	Neonatal outcome in 29 pregnant women with COVID-19: A retrospective	Wu et al.	China	29	2	0	27	5	The findings suggest that intrauterine or intrapartum transmission is	216

Number of study	Title	Authors	Country	N of woman with SARS-CoV-2	Total neonates vaginal birth	Infected neonates vaginal birth	Total neonates caesarean birth	Infected neonates caesarean birth	Author's conclusion	Ref.
	study in Wuhan, China								possible and warrants clinical caution and further investigation.	
50	Coronavirus disease 2019 among pregnant Chinese women: case series data on the safety of vaginal birth and breastfeeding	Wu Y. et al.	China	3	1	0	2	0	This case series indicated that vaginal delivery may be a safe delivery option.	217
51	Clinical Manifestation and Neonatal Outcomes of Pregnant Patients With Coronavirus Disease 2019 Pneumonia in Wuhan, China	Xu et al.	China	23	5	0	18	0	No evidence indicated that pregnant women may have fetal infection through vertical transmission of SARS-CoV-2.	218
52	Coronavirus disease 2019 in pregnant women: a report based on 116 cases	Yan et al.	China	99	14	0	85	0	There is no evidence of vertical transmission of	219

Number of study	Title	Authors	Country	N of woman with SARS-CoV-2	Total neonates vaginal birth	Infected neonates vaginal birth	Total neonates caesarean birth	Infected neonates caesarean birth	Author's conclusion	Ref.
									SARS-CoV-2 infection.	
53	Effects of Severe Acute Respiratory Syndrome Coronavirus 2 Infection on Pregnant Women and Their Infants	Yang et al.	China	23	5	0	18	0	There was no distinct evidence of vertical transmission from mothers with SARS-CoV-2 to their newborns.	220
54	Pregnant women with COVID-19 and risk of adverse birth outcomes and maternal-fetal vertical transmission: a population-based cohort study in Wuhan, China	Yang et al.	China	65	13	0	52	0	The data provide little evidence for maternal-fetal vertical transmission of SARS-CoV-2.	221
55	Clinical features and outcomes of pregnant women suspected of coronavirus disease 2019	Yang et al.	China	13	4	0	9	0	The clinical symptoms and laboratory indicators are not obvious for	222

Number of study	Title	Authors	Country	N of woman with SARS-CoV-2	Total neonates vaginal birth	Infected neonates vaginal birth	Total neonates caesarean birth	Infected neonates caesarean birth	Author's conclusion	Ref.
									asymptomatic and mild COVID-19 pregnant women.	
56	Severe Acute Respiratory Syndrome Coronavirus 2 (SARS-CoV-2) Infection During Pregnancy In China: A Retrospective Cohort Study	Yin et al.	China	17	4	0	13	0	There was no evidence of vertical transmission during pregnancy with SARS-CoV-2 infection.	224
57	Update on clinical outcomes of women with COVID-19 during pregnancy	Zeng et al.	Chuna	16	4	0	12	0	There was no distinct evidence of vertical transmission from mothers with SARS-CoV-2 to their newborns	225
58	Severe acute respiratory syndrome coronavirus	Zhang et al.	China	18	1	0	17	0	Vertical transmission of	226

Number of study	Title	Authors	Country	N of woman with SARS-CoV-2	Total neonates vaginal birth	Infected neonates vaginal birth	Total neonates caesarean birth	Infected neonates caesarean birth	Author's conclusion	Ref.
	2(SARS-CoV-2) infection during late pregnancy: a report of 18 patients from Wuhan, China								SARS-CoV-2 was not detected.	
59	Clinical analysis of 10 neonates born to mothers with 2019-nCoV pneumonia	Zhu et al.	China	9	2	0	7	0	Vertical transmission of SARS-CoV-2 was not detected.	227
